# Progress of the Medical Sciences

**Published:** 1914-06

**Authors:** 


### Progress of the Medical Sciences.

### MEDICINE.

**Splenomegaly with anæmia.**—Osler has recently referred to the "dust-heap" of diseases which exist under this heading, and which are being gradually separated out into distinct syndromes. A curious thing about them is the number of different affections which are cured or relieved by splenectomy. If we allow that true splenic anæmia is due to a poison arising in the spleen, then it is easy to see that removal of the spleen may remove the source of the mischief; but since it is clear that certain diseases due to syphilis and other causes do actually benefit by splenectomy, we must either conclude that these enlarged spleens produce secondary evils, or become culture grounds for the special poison concerned.

A most interesting announcement has been made by A. G. Gibson that he had found in a typical case of true splenic anæmia clear evidence of a dense growth of a streptothrix in the spleen. Stained by Wheal and Chown's method, black threads and masses could be seen. The threads were segmented and not branching, but breaking off at the ends into bacillary forms. It was certain that the threads were not fibrin, fibrous, or elastic tissue. Marked fibrosis of the trabeculæ and pulp, especially around the veins, was present. Similar appearances of the streptothrix type were found in five other diseased spleens which he examined. Though considerable doubt has been thrown on the interpretation of these cases, it is probable that ere long some organism may be detected in such spleens. On May 8th Gibson showed a thick bacillus, non-motile and Gram negative, which he had isolated from another case of Banti's disease.

The discussions at the Royal Society of Medicine [1] and the work of certain German surgeons have shown remarkable results from splenectomy in many unexpected conditions. In 1895 Spanton could only collect a hundred cases in the last four hundred years, but of late it has been performed frequently. Taking Osler's classification provisionally, we find the following conditions are already separated :—

1. True splenic anæmia is usually marked by leucopenia and a low-colour index. It gradually progresses for some years, and is attended by hemorrhages, which may become fatal. The splenic enlargement precedes the anæmia, and the condition is later on often complicated with hepatic cirrhosis, jaundice and ascites, when it is called Banti's disease. If iron, salvarsan and X-rays fail, recourse is not infrequently had to splenectomy, which has a mortality of perhaps 12.5 per cent., but in the Banti state a higher rate must be expected.

2. Splenomegaly of the Gaucher type begins most often in children under twelve, and may attack several in one family. The spleen may reach a huge size, the average being seven pounds, and the liver too becomes very large. There is leucopenia, a brownish yellow pigmentation of the face and hands, patches of conjunctival thickening, epistaxis, bleeding from the gums and into the skin, but life may be prolonged twenty or thirty years. Erdman and Moorhead [2] have recently done splenectomy with great success in one case, and report ten others with eight recoveries.

3. Splenomegaly with acholuric jaundice is also a family disease, and sometimes hereditary. Chauffard showed that there is a remarkable fragility of the red cells in saline solutions. The duration is long, indeed it hardly shortens life, but patients are helpless invalids. The anæmia may be extreme, but sometimes leucocytosis exists. It appears that Spencer Wells's case of splenectomy in 1887 was of this type. The patient is still alive and in good health, though the fragility of the red cells continues, as sometimes happens. Essex Wynter and H. Thursfield [3] showed two cases of complete restoration to health after splenectomy, while Whipham brought forward a father and three children

[1] *Proc. Roy. Soc. Med.*, 1914, vii., Clin. Sect., p. 73 *et seq.*

[2] *Am. J. M. Sc.*, 1914, cxlvii. 213.

[3] *Proc. Roy. Soc. Med.*, 1913, vii., Clin. Sect., p. 82, *et. seq.*

all suffering from it since a febrile attack three years ago. Sir Bertrand Dawson reported successful operations in three young adults of probably the same type, but noted the presence of gall-stones and biliary sand, which he removed at the same operation. It may be interesting to notice that Aschoff [1] has recently shown that gall-stones do not always pre-suppose the occurrence of inflammatory catarrh of the gall-bladder, and that a cholesterin stone may be formed without any such condition if cholesterin is present in excess in the blood. This excess occurs in pregnancy, Bright's disease, jaundice, diabetes and, perhaps, typhoid. The amount may rise from the normal of 1.6 grm. per litre to 4.2.

4. Splenomegaly due to syphilis of the liver may closely simulate true splenic anæmia, the splenic features over-shadowing the hepatic, and of this Osler gives two or three instances. Anti-syphilitic treatment may have little effect. Here, too, splenectomy has been done with success by Coupland and others. The uncertainty of diagnosis can, of course, be genearlly cleared up by the Wassermann test. In intractable cases of pseudo-leukæmia of the von Jaksch type splenectomy has been recommended, and French showed a case of complete recovery after the operation, but doubt has been thrown on his diagnosis. There is often here a lymphocytosis and many nucleated red cells.

5. Splenomegaly due to infective endocarditis may for a time be difficult to distinguish, but the short duration, persistent albuminuria or hæmaturia would be of assistance. Still it is remarkable that an exact picture of splenic anæmia with leucopenia may be present.

6. Splenomegaly from obstructive thrombosis is also said to exactly resemble splenic anæmia at times, and there may be a history of injury or strain pointing to the cause of the thrombosis. On the other hand, as Rolleston [2] remarks, thrombosis is not infrequently a late result of splenic anæmia, and Rodman and Willard [3] confess that experimental attempts to produce the disease by ligaturing the splenic or portal veins have entirely failed.

7. Tropical splenomegaly due to Kala-azar especially of the type seen in children on the shores of the Mediterranean, may closely imitate the disease, and of course in some cases of malaria the same difficulty may be felt, but generally the parasites can be discovered.

8. Splenomegaly secondary to cirrhosis of the liver may

[1] *Glasgow M. J.*, 1914, lxxxi. 108; also *Quart. J. Med.*, 1914, vii. 221.
[2] *Practitioner*, 1914, xcii. 470.
[3] *Ann. Surg.*, 1913, lviii. 601.

occasionally show very little symptom of the liver trouble. Indeed, "Naunyn and his followers regard latent cirrhosis as the real explanation of splenic anæmia." An Egyptian type, commencing with cirrhosis and marked fever, is otherwise indistinguishable from splenic anæmia, and here splenectomy at an early stage arrests the disease.

9. Finally, Eppinger and several German surgeons[1] have recently performed splenectomy for pernicious anæmia. Six cases are reported since August, 1913, all of which have improved, though it is too early to speak of a cure. In every instance the operation was followed by a great increase of nucleated red cells. It was to be expected that the removal of an enlarged spleen would reduce the facility with which the red cells are hydrolised, as that result has been observed in other conditions. On the other hand, no evidence has been given to show that anything but harm can result from splenectomy in the leukæmias.

**Anaphylaxis.**—McIntosh[2] in an interesting paper brings forward much evidence to show that the ordinary definite series of symptoms which occurs in the course of a fever are the results of anaphylaxis. These manifestations have hitherto been mysterious and unaccountable. Anaphylaxis is, he points out, a state of hypersensibility to some albuminous substance either existing normally or as the result of previous injections. It is the albuminous serum injected and not the toxin which plays the active part. Still it is an immunity reaction. Allergie is the local reaction of hypersensitive tissues. An incubation period of 6 to 12 days is usually required for the production of hypersensitivity.

In fevers the introduction of the antigen both sensitises the tissues and causes the development of antibodies during the incubation period. Endotoxins being formed act on the sensitised cells. Hence the rash in measles, scarlatina and small-pox, and in serum fever. In revaccination for small-pox after a short period the papule and areola are much more quickly developed from the hypersensitiveness of the patient.

Tuberculous patients show an extraordinary sensitiveness to Koch's tuberculin, but after repeated injections become desensitised, and until this is done immunisation with large doses cannot be carried out. The von Pirquet and Calmette reactions show local reactions from anaphylaxis, though the entire process is not yet explained.

In syphilis reinoculation in the early incubation period gives an abnormal papule with quicker development. In the secondary stage the patient is insusceptible, and no lesion

[1] *Surg., Gynec., and Obst.*, 1914, xviii. 243.
[2] *Quart. J. Med.*, 1914, vii. 272.

follows. The rash is due to the destruction of spirochætes in the skin and the toxins set free there. The tissues in the tertiary stage are hypersensitive, a skin reaction like von Pirquet's, called the Luetin test, is positive, and the gumma is an anaphylactic reaction of lymph vascular tissues. Parasyphilitic lesions are the response of hypersensitive nerve tissues with no power of regeneration. Hay fever is the reaction of mucous membranes hypersensitised by pollen proteid. Urticaria is an expression of hypersusceptibility of tissues to foreign proteins. The sensitiveness is either constitutional or acquired. Eclampsia is due to the reabsorption of placental villi. The mother is sensitised to the foreign or fœtal part of the placental protein. Indeed, one author suggests that parturition is itself an anaphylactic phenomenon due to the excessive absorption of some product of the child, and some evidence has been adduced in favour of this view.

Though we cannot at once accept all the teaching of this school, the theories do offer a reasonable explanation of numerous obscure phenomena, and are worthy of consideration.

**Poliomyelitis.**—From the prolonged study which has been given to the mode of transmission, some few facts have been obtained.

The virus with which Flexner and others[1] have been able to reproduce the disease in monkeys was for some time thought to be ultramicroscopic, like those of yellow fever and several other diseases, but it turns out to be a minute globoid body, like that found in rabies, and it passes through Berkefeld filters. Noguchi, employing his method of cultivating spirochætes, has grown them in ascitic fluid, with a fragment of sterile kidney tissue. Oxygen is excluded by a layer of liquid paraffin, and after five days' incubation an opalescence appears. The globoid bodies are arranged in chains or groups, and stained by Gram or Giemsa's method. They are constantly found in the central nervous system of infected persons, and the disease can be reproduced from them. Amoss also found them in blood films from infected monkeys during the acute stage. The virus, however, shows a preference for nerve tissue. It affects the whole nervous system, especially the motor neurons of the cord and brain, and exists even in the sympathetic ganglia. It is apparently soon swept out of the blood, which as a rule is incapable of conveying the disease to other animals, except for a short time after large quantities of the virus have been injected into it. Indeed, the injection of moderate doses fails to produce symptoms, though they regularly follow injections into the brain or cord. The cerebrospinal fluid, in like manner, fails to convey the disease, and when

[1] *J. Exper. Med.*, 1913, xix. 205, 411, *and* 1914, xx, 212.

taken from convalescents, rarely contains the neutralising bodies.

The virus is highly resistant, since it remains active in glycerine for two years, or for a year in 0.5 per cent. carbolic, and after freezing for some weeks, though it is easily killed by heating to 50° C. for half an hour, by permanganate, menthol, or peroxide. It enters the human body chiefly through the nasal mucous membrane, rarely if at all in food. Petterson and others found it in the nasal secretion of infected persons and their healthy companions, who may act as "carriers," and also in the dust of rooms where infected persons live, and on their handkerchiefs. Convalescents may remain infectious for months or years, though probably the majority cease to be so in four weeks. There is some evidence, too, that the disease may exist in rabbits or other animals under another form, but regain its old power when returned to man or monkeys. There are great difficulties in deciding as to the actual mode by which infection is transmitted. It is a rural rather than a city disease, and rarely spreads in an institution where patients are in close contact. Rosenau apparently showed that it could be conveyed by the stable fly Stomoxys Calcitrans, but Sawyer and Herms[1] failed to confirm this; besides, the blood is rarely infective, as we have seen. The bug, too, has been credited with its transmission, but on the whole there is little reliable evidence in favour of insect or animal carriers. Besides the large numbers of persons who are immune there are many abortive cases. Wickham estimated the latter at from one-fourth to one-half of the whole. By various tests they can be shown to be real instances, though paralysis is absent, and they certainly can transmit the infection during their convalescence. It has been remarked that outbreaks come in waves once in two or more years. Thus during 1911 and 1912 there were numerous epidemics in Great Britain, and comparatively few cases since. In Sweden, after a quiet period, 3,800 cases appeared in 1911; and since about 16 per cent. usually die, and the majority of those who recover are crippled for life, the disease is most serious. As regards the periodicity, it is possible that climatic influences are important, or that most of the susceptible persons are rendered immune by unrecognised abortive attacks, but neither theory is probable. Flexner[2] has suggested that the organism itself has a cycle of variations in virulence as time goes on, which is the cause of the epidemic waves. When it was passed through a series of monkeys for some years a rise of potency took place, and lasted for perhaps three years, and then a gradual fall to the original state took place.

[1] *J. Am. M. Ass.*, 1913, lxi. 461.
[2] *J. Exper. Med.*, 1914, xix. 203.

which is certainly suggestive. Colliver[1] calls attention to a symptom in the preparalytic stage which may be of use. It is a peculiar brief tremor or twitching affecting one or more muscles or limbs, and recurring at varying intervals. Sometimes there is a slight cry, and sometimes during a tremor the child seems to lose consciousness for a few seconds, though there is never definite coma. There is some doubt, however, whether this tremor does not also occur in cerebrospinal fever. The spinal fluid after the first week is clear, colourless, reduces Fehling, and shows no bacteria, but only mononuclear cells and much globulin, which is in marked contrast with that of cerebrospinal fever. The fluid during the first days of the attack indeed may be cloudy, and show polymorphic cells. In typical cases there is a stage of incubation of three or four days, a febrile stage of one to seven days, with pain on passive movement of the limbs, and lastly the stage of paralysis, cerebral, cerebellar or spinal, though in some cases no symptoms appear before the paralysis. This may go on increasing for a week, and cause death by respiratory failure. There is finally a period of improvement, which under treatment may continue for years.

**Artificial Pneumothorax.**—The idea of treating phthisis by opening the pleura and immobilising the lung is an old one. It began with suggestions made by Carson (1821) and Ramadge (1834),[2] but was put into practice by Cayley (1885), Potain (1885), Forlanini (1888), Murphy (1898), Lemke (1899), and Brauer (1905). Since then it has been extensively employed.

At the present time the primary operation is either an incision or a puncture. The latter is generally chosen in this country, though in difficult cases where many adhesions exist an incision may be necessary. The method usually employed in Germany and Switzerland is by an incision. Local anæsthesia in any case must be carried out thoroughly to prevent shock, and an elaborate apparatus for introducing nitrogen is always used, by which the exact intrapleural pressure can be seen and regulated, and the amount of nitrogen passed into the pleural cavity is known. As a precaution against the accidental introduction of nitrogen into a vein [either by puncturing it or by exposing an opened vein to a body of gas at high pressure, and in this way causing embolism, with possibly fatal results.] E. Rist, Morriston Davies and others first fill the apparatus with oxygen. If this enters a vessel it combines with the hæmoglobin, and is absorbed harmlessly. After 50 or 100 cc. have been thus introduced nitrogen is turned on. Authorities differ as to the amount of nitrogen which should be injected on the first occasion, some giving 100 cc. and others a larger

[1] *J. Am. M. Ass.*, 1913, lx. 813.
[2] *Quart. J. Med.*, 1913, vi. 259.

amount. The intrapleural pressure, as shown by the manometer, is carefully watched to prevent any excess. A positive pressure of 1 to 3 mm. of mercury is sufficient to keep the lung compressed, when no adhesions exist. To maintain this collapse permanently, as the resistance decreases it is necessary to refill the pleura with nitrogen from time to time. At first this is done by a simple puncture every day or two, but later on once in every month or six weeks. X-ray examinations show the compression and general condition of the lung on each occasion. Among the accidents which may occur, a pleural reflex with collapse tachycardia and delirium, practically never arises if complete local anæsthesia of the tissues and pleura is attained before the puncture is made, and if we avoid the rapid production of a high positive pressure.

Deep emphysema occasionally gives pain and discomfort. Indeed, pain is not infrequently felt if too high pressure is produced, but is not lasting. Displacement of the heart must be guarded against by using the manometer and fluoroscope. Pleural effusions frequently occur, and are of no consequence except in rare cases, where if the pressure is too high it may be necessary to withdraw some of the gas. Empyema may occur from a fresh infection, or from wounding a diseased lung by using a sharp trocar instead of a blunt one.

What patients are suitable for this treatment? Cases where unilateral lesions exist are preferable, but slight disease in the other lung is no bar to the operation. Amrein and Lichtenhahn, [1] of Arosa, urge that the greatest care be taken to avoid cases of active disease in the other lung. Other writers say there should be at least two-thirds of the other lung in a healthy state. The acting lung sometimes improves remarkably after the more diseased one has been collapsed, though occasionally it fails to respond, and the gas has to be withdrawn. Even laryngeal trouble is benefited by the cessation of cough. Amyloid kidneys and general miliary tuberculosis counter-indicate the treatment. Hæmoptysis is quickly stopped by the operation. Indeed, no other method of controlling it is so effective. As much as 500 cc. of nitrogen may be introduced at one sitting to stop severe bleeding.

How far should this treatment be applied to early cases in place of or with sanatorium treatment? Certainly some recover wonderfully well and quickly. H. Morriston Davies, [2] indeed, holds that no progressive case is too early, and points out that lungs with very little disease respond more readily to the treatment, and that failures from general adhesions are fewer. Again bronchiectasis, chronic pneumonias, as well as

[1] *Ibid.*, p. 487.
[2] *Brit. M. J.*, 1914, i. 897.

phthisis, have been claimed by some authors as giving excellent results. However, in bronchiectasis a permanent cure is not to be expected, but only great improvement as long as compression is continued.

It is acknowledged that in unilateral phthisis, when fever, cavities, and much expectoration are present, the operation causes a rapid disappearance of the symptoms, a gain of weight and general improvement, though if the pressure is allowed to fall too soon a relapse may take place. How long then should compression be kept up? This is difficult to answer. Until the tuberculous lesions are cured, even though bacilli are absent from the sputum, it is better to go on refilling with nitrogen. In many cases there is complete healing. Cavities are obliterated, their walls are pressed together and unite. Abundant fibrous tissue grows up around the bronchi and vessels where ulceration has taken place, and the same tissue encloses the small pneumonic and tuberculous foci. The liquid discharge is squeezed out of the collapsed lung, and, most remarkable of all no fresh tubercle has ever been found in it.

Discussion has arisen as to whether this great fibrous hypertrophy is the result of the tuberculous toxin. It has, however, been found in absolutely healthy lungs when collapsed. The question is not yet settled.

We may add that in the ordinary removal of serous effusions from the pleura H. Morriston Davies carries out the process by alternately withdrawing a little fluid, and introducing an equal quantity of oxygen at a higher level till the whole of the fluid is removed. He claims that much more fluid can be removed, and that pain shock and the chance of œdema of the lungs is prevented. The slow absorption of the oxygen also causes a better expansion of the lung.

George Parker.

### SURGERY.

**Prostatism** is a term used to designate the group of symptoms resulting from a diseased prostate which produces interference with the complete emptying of the bladder, changes in the genito-urinary tract above, and ill-health of the patient. In writing on this subject, Wade[1] points out that supra-pubic prostatectomy for prostatic dysuria is associated with a certain mortality, and sometimes followed by disagreeable complications, so that in many cases the operation is neither safe nor certain.

There is an early period of the disease when these unpleasant sequences are minimised, but a large number of patients—

[1] *Ann. Surg.*, 1914, lix. 321.

particularly those of the hospital class—do not come to operation until considerable damage has occurred to the bladder, ureters and kidneys, and in these cases the danger of operation is by no means negligable. Wade quotes the statistics of various hospitals, and says that according to the published statements of one of the largest hospitals in America, out of 164 cases operated on during ten years 54 died, a mortality of 35.4 per cent. In St. Thomas's Hospital during the five years 1906–10, 69 cases were treated by supra-pubic prostatectomy, of which 14 died, a mortality of 20.3 per cent. He also quotes Page, who says that the mortality after the operation of supra-pubic prostatectomy for adenomatous enlargement of the prostate in St. Bartholomew's, University College, Westminster and Middlesex Hospitals, during the period 1906–10, was 21.5 per cent. Page has collected the cases that were admitted to St. Thomas's Hospital[1] and tabulated them according to the treatment received. The total number of cases was 132, and the mortality 21.7 per cent. Those treated by catheterisation had a mortality of 22.7 per cent., those treated by supra-pubic drainage a mortality of 20 per cent., and those by supra-pubic prostatectomy a mortality of 20.3 per cent. Individual operators can show a much lower mortality than obtains in these general statistics, but Wade considers that the figures quoted of the mortality attending prostatectomy in general hospitals has not shown in recent years that degree of reduction we might have been led to expect.

An analysis of 68 fatal cases goes to show that the commonest cause of death after supra-pubic prostatectomy is septic absorption, arising out of the wound inflicted, and acting upon patients with weakened power of resistance owing to the prolonged disease. The dangers peculiar to supra-pubic prostatectomy are the wounding of the space of Retzius and the consequent risk of septic pelvic cellulitis, pulmonary infection with hypostatic congestion and pneumonia, and the difficulty of efficient drainage of the bladder and prostatic bed so as to prevent septic absorption from the septic stagnant urine that bathes the raw surfaces.

We may consider how far these risks are avoidable by adopting other means of treating the disease, the choice lying between catheter life, preliminary supra-pubic cystotomy and perineal prostatectomy. Catheter life may be dismissed as justifiable only in the inoperable cases. Preliminary supra-pubic cystotomy is performed by some on the grounds that it permits of an improvement in the patient's condition prior to the removal of the growth, and improves the condition of the septic bladder, but Wade thinks the value of it is doubtful.

[1] *St. Thomas's Hosp. Rep.*, 1912, xxxix. 135.

On the other hand, Deaver[1] gives it as his opinion that a two-stage operation, while not ordinarily advisable, may succeed in the case of enfeebled patients with severely infected urinary tracts. The unavoidable risks peculiar to perineal prostatectomy are the greater length of time occupied by the operation and the risk of infection of the pelvic cellular tissue planes, owing to the opening of the lymph paths when the space of Dénonvilliers is opened into. This latter risk is, however, practically avoided by the "packing" which is carried out after operation. Though the supra-pubic route is generally preferred because of its greater ease of performance, Deaver[2] thinks the perineal route is best (1) in cases of carcinoma when lines of cleavage have been obliterated, (2) in tuberculosis of the prostate, (3) in the small sclerotic prostates of chronic prostatitis or fibrous hypertrophy.

The following form the basis of Wade's conclusions on the subject: (1) Three outstanding varieties of disease lead to prostatism — (*a*) prostatic hypertrophy or chronic lobular prostatitis; (*b*) prostatic fibrosis or chronic interstitial prostatitis; (*c*) prostatic carcinoma. (2) The first is by far the commonest cause of prostatism and was present in 82 per cent. of the specimens examined. (3) Chronic lobular prostatitis is a senile hyperplasia, an aberrant growth of tissues that is not the result of the appearance of an independent new growth, but is liable to develop into the same. (4) Chronic lobular prostatitis in the majority of cases produces prostatic hypertrophy. (5) It virtually always develops in the middle lobe, and is almost uniformly confined to the middle and lateral lobes. (6) The gland in consequence undergoes changes that usually permit of its easy removal by supra-pubic prostatectomy. (7) Chronic lobular prostatitis may develop in and be confined to the anterior lobe. (8) Chronic lobular prostatitis may cause prostatism without enlargement of the organ, intravesical herniation or complete false capsule formation. (9) In these cases the performance of supra-pubic prostatectomy is difficult and dangerous. (10) The successful performance of supra-pubic prostatectomy depends on the presence of an advanced type of prostatic hypertrophy due to chronic lobular prostatitis. (11) The clinical recognition of this is frequently very difficult. (12) When a patient with this advanced type of disease is operated on, his urinary tract and general health have usually suffered serious damage from the disease. (13) It is, therefore, unjustifiable to delay operation in an early case of chronic lobular prostatitis in order to permit of the gland undergoing those hypertrophic changes that facilitate its easy removal by supra-pubic prostatectomy. (14) The mortality attending

[1] *Ann. Surg.*, 1914, lix. 360. [2] *Loc. cit.*

supra-pubic prostatectomy is mainly due to the impaired health of the patient prior to operation. (15) The actual cause of death in such cases is usually a local affection arising out of the wound inflicted. (16) The operation of supra-pubic prostatectomy by blind enucleation is unsuitable in cases of prostatism due to other causes than advanced chronic lobular prostatitis. (17) Perineal prostatectomy is a most suitable operation in such cases. (18) Perineal prostatectomy permits of the removal of the disease when its presence is diagnosed in all cases. It is, therefore, at present the operation that offers the best prospect of further advance in the treatment of prostatism. (19) For its successful performance an accurate knowledge of the anatomical structure and relationships of the prostate are necessary, as well as an understanding of the pathology of the disease. (20) The supra-pubic transvesical method of prostatectomy by visual dissection offers the prospect of developing into a method of treating prostatism that may ultimately warrant its adoption in a large number of cases. (21) Chronic interstitial prostatitis is best treated by division and removal of the constriction by the trans-urethral route. (22) Prostatic carcinoma may be in an early case clinically indistinguishable from hypertrophy due to chronic lobular prostatitis. (23) This fact is, therefore, a further reason for operation in all such cases. (24) Prostatic carcinoma when recognised clinically may be successfully treated by excision of the gland in suitable cases.

The **carotid body** is assuming a position of some importance, and Collison and Mackenty[1] give an account of what is known of this organ or gland in dealing with tumours of the carotid body. The structure is about a quarter of an inch long and an eighth of an inch wide, and is situated in the bifurcation of the carotid artery. Many older observers have supposed that the body is derived from the third or fourth branchial cleft or from the perithelium of the carotid arteries, but the authors of the paper referred to favour the view of Zuckerkandl, Stilling, and others that it belongs to the chromaffin system. Many of the cells of which it is composed stain brown with neutral salts of chromic acid, just as is the case with similar cells found in the medulla of the supra-renals, in the pituitary body and in the ganglia of the sympathetic nervous system. The carotid body may, therefore, be regarded as derived from the cervical sympathetic system.

Little is known of the physiology of the carotid body, and experimental evidence is not in favour of the existence of an

[1] *Ann. Surg.*, 1913, lviii. 740.

important internal secretion, but possibly its function may be connected with trophic stimuli in body development.

Our pathological knowledge of the "body" is limited to tumours, of which about sixty have been reported. The causation of the tumours is obscure. The carotid body is supposed to reach full development between 20 and 30 years of age, and then atrophies or goes on to tumour formation, and thus we find that the greatest number of cases occurs from 20 to 50 years.

These tumours generally have a long history of slow growth, and during this time they have the characteristics of benign neoplasms, being encapsuled and showing no tendency to return if removed, though complete removal is difficult because of their position. Later on they may grow more rapidly and have the characteristics of tumours of slight malignancy, recurrences and metastases may occur, but the cachexia of malignant growth is not present. They have been usually diagnosed as endotheliomata or peritheliomata.

At first the patient complains only of the deformity, but later on there are symptoms of involvement of the cranial and sympathetic nerves, such as cough, dysphagia, or deafness. As the tumour grows more and more structures are involved, the mobility is less, the recurrent laryngeal nerve is paralysed, and the tumour may bulge into the pharynx.

Tumours of the carotid body must be differentiated from lymphadenitis, carcinomatous glands, lymphosarcoma, lipoma, fibroma, aneurism, branchial cysts, gumma, Hodgkin's disease, aberrant thyroid and Bezold's perforation of the mastoid. In many cases a diagnosis can only be made after submitting a portion of the tumour to the microscope.

A diagnosis is rarely made in the early stage, but an operation is attempted under some mistaken diagnosis, and then the true nature of the growth is discovered. The tumour may be dissected away from the bifurcation if it is not closely adherent to the arteries; but if the tumour surrounds the blood-vessels, is closely adherent to the wall, and other structures are included in the growth, then it is unwise to attempt to dissect the mass away, unless the surgeon is prepared to face the risk of removing tumour and arteries together. This involves ligation of the common, internal and external carotid arteries, and probably also of the jugular vein, and the attendant dangers of injuring the pneumogastric, hypoglossal and other nerves. In many of these cases it would be wiser to decline removal of the tumour and close the wound.

JAMES SWAIN.

